# SERS-Active Pattern in Silver-Ion-Exchanged Glass Drawn by Infrared Nanosecond Laser

**DOI:** 10.3390/nano10091849

**Published:** 2020-09-16

**Authors:** Ekaterina Babich, Vladimir Kaasik, Alexey Redkov, Thomas Maurer, Andrey Lipovskii

**Affiliations:** 1 Institute of physics, nanotechnology and telecommunications, Peter the Great St. Petersburg Polytechnic University, Polytechnicheskaya 29, 195251 St. Petersburg, Russia; vkaasik@yandex.ru (V.K.); lipovskii@mail.ru (A.L.); 2 Sector of optics of heterogeneous nanostructures and optical materials, Alferov University, Khlopina 8/3, 194021 St. Petersburg, Russia; 3 Laboratory of structural and phase transformations in condensed media, Institute of Problems of Mechanical Engineering RAS, Bolshoy pr. V. O. 61, 199178 St. Petersburg, Russia; red-alex@mail.ru; 4 Light, Nanomaterials, Nanotechnologies (L2n), Université de Technologie de Troyes & CNRS ERL 7004, rue Marie Curie 12, CS 42060, 10004 Troyes CEDEX, France; thomas.maurer@utt.fr

**Keywords:** silver nanoparticles, SERS, glass, ion-exchange, nanosecond laser

## Abstract

The irradiation of silver-to-sodium ion-exchanged glass with 1.06-μm nanosecond laser pulses of mJ-range energy results in the formation of silver nanoparticles under the glass surface. Following chemical removal of ~25-nm glass layer reveals a pattern of nanoparticles capable of surface enhancement of Raman scattering (SERS). The pattern formed when laser pulses are more than half-overlapped provides up to ~10^5^ enhancement and uniform SERS signal distribution, while the decrease of the pulse overlap results in an order of magnitude higher but less uniform enhancement.

## 1. Introduction

It is recognized, glasses with silver nanoparticles (SNPs) on their surface are beneficial in surface-enhanced Raman spectroscopy (SERS) employed in chemo- and bio-sensors and in microfluidic platforms for screening, monitoring, and quantitative analysis of a molecular probe [1,2]. Moreover, their biomedicine-related implementation includes antibacterial, antifungal, and antiviral applications [3,4]. The attractiveness of multicomponent glasses as substrate material is their well-developed technology and low price, while the main advantages of SNPs are selective toxicity and the highest among other noble metals quality factor of surface plasmon resonance (SPR) from visible to infrared spectral range [5]. Mentioned applications require SNPs to be placed on the glass surface, which can be implemented by various methods. The latter includes depositing thin silver films either followed by their annealing [6] or combined with different lithographic techniques [7,8]; depositing nanoparticles using laser ablation of silver targets [9]; sedimentation of SNPs from solutions [10], e.g., resulting from the laser irradiation [11]; thermal [12] or reactive reduction [13] of silver ions in silver-ion-exchanged glasses followed by SNPs precipitation on their surface by out-diffusion. One more developing approach to the formation of SNPs is the irradiation of silver-ion-exchanged glasses by ultra-short laser pulses of different wavelengths in the visible [14,15], IR [16,17], and UV [18,19] spectral ranges or by continuous short-wavelength lasers [20,21]. Contrary to others, this technique allows direct formation of SNPs and “drawing” of a given 2D pattern filled with SNPs. Note, only in the case of irradiation by either continuous [22] or femtosecond [23] laser it was demonstrated that SNPs form on the glass surface, while there are no studies, to our knowledge, related to SNPs distribution in other cases.

Overall, in the listed methods, SNPs are formed directly on the surface and while it is beneficial for, e.g., SERS-activity, fabricated substrates are one-use and suffer from silver sulfidation and oxidation while stored, if non-protected [24].

In this paper, we studied formation and distribution of SNPs in a silver-ion-exchanged glass after its nanosecond IR laser irradiation. We demonstrated that pattern from SNPs repeats laser beam trajectory while SNPs formation happens in the subsurface glass layer. Finally, SERS-activity of SNPs in the laser-irradiated regions subjected to chemical etching was confirmed. Thus, the study demonstrates the route to fabricate SERS substrates of a given design, which have potential for multiple usages: sequential etching of different parts of the substrate uncovers SNPs which are not affected by interaction with the environment.

## 2. Experimental

We used a soda-lime glass (Agar Scientific Ltd., Stansted, UK) containing 14.3 wt.% of sodium oxide. A 1-mm thick slide was silver-ion-exchanged for 20 min in the melt of (AgNO_3_)_5wt.%_(NaNO_3_)_95wt.%_ (LenReactiv, Saint Petersburg, Russia) at 325 °C. The maximal depth of silver ions penetration (zero concentration level) and the maximal silver oxide concentration at the glass surface were evaluated following the method of Ref. [25] as ~8 μm and ~10.5 wt.%, respectively. Thus, more than 70% of sodium in the glass was replaced by silver. Generally, higher content of silver ions in the glass results in higher number of SNPs. However, following increase of AgNO_3_ content in the melt demonstrated only a weak increase in silver ions concentration in the glass, while using of highly concentrated or pure AgNO_3_ melt is restricted because of its decomposition.

We irradiated the silver-ion-exchanged glass with a Nano L Nd:YAG laser (wavelength of 1.06 μm, Litron Lasers Ltd., Warwickshire, UK) to form SNPs. The laser provided 6-nanosecond long pulses with the maximal energy of 14 mJ, whose frequency could be varied from 0.1 to 30 Hz. Particularly, the laser beam was focused on the glass surface to a spot size of ~250 μm in diameter and some regions (“lines”) were irradiated using the computer-driven platform MT3/M-Z8 3D (Thorlabs Inc., Newton, NJ, USA) that moved the glass slide in the plane perpendicular to the laser beam at the speed of 200 μm/s. Thus, a line was a row of either individual irradiated spots (0.1–1 Hz) or overlapped spots. In experiments, starting from 5.6 mJ pulse energy, intense flashes of light were observed under the irradiation of the silver-ion-exchanged glass. This pulse energy also corresponded to yellowish coloration of the sample. Further increase in pulse energy resulted in darkening of the sample and finally destruction of the surface at 7.5 mJ. Therefore, we studied lines irradiated at 6.3 mJ and 2 Hz, and 5.6 mJ and 3.5 Hz, the overlap being ~50% and ~70% of a single irradiated spot area, respectively.

We measured optical absorption in the laser-irradiated regions using a homemade setup with QE65 Pro spectrometer (OceanOptics Inc., Dunedin, FL, USA), 50 μm collecting optical fiber and 20×/0.4 objective (~50 μm collection spot). The surface of the irradiated lines was also characterized with optical profilometer Wyko NT9300 (Veeco Instruments Inc., Plainview, NY, USA) and scanning electron microscope JSM 7001F (JEOL, Tokyo, Japan) with energy-dispersive X-ray spectroscopy (EDS) system INCA PentalFETx (Oxford Instruments, Abingdon, UK).

Formed structures were tested as SERS substrates using Raman spectrometer Alpha 300R (WITec, Ulm, Germany) equipped with 532 nm laser and 10×/0.25 objective (~3 μm collection spot). We chose 1,2-di(4-pyridyl)ethylene (BPE, Sigma-Aldrich Co., St. Louis, MO, USA) as a probe molecule, which is commonly used in case of 532 nm excitation because of its non-resonant behavior [26,27]. The spectra were collected in the center of the dried droplet (4 μL) of BPE water solution (10^−4^ M) along the laser-irradiated lines on the glass. We mapped the regions 100 × 3 μm^2^ with 5 μm step, the integration time was 3 s per point, the laser power ≈0.4 mW. To evaluate the enhancement factor (EF), we collected Raman spectrum from BPE crystallites, which were placed on the non-irradiated and non-ion-exchanged glass surface. The spectrum was averaged over 20 measurements across different crystallites, the integration time was 3 s per measurement and laser power ≈0.4 mW. The average size of the crystallites was ~25 μm. The background in all of the measured spectra was subtracted using MathLAB function [28] (MathLAB 9.7, The MathWorks Inc, Natick, MA, USA) based on the algorithm described in Ref. [29].

Additionally, we performed chemical etching of the laser-irradiated samples in HF(5 μL):NH_4_F(5 g):H_2_O(40 g) (LenReactiv, Saint Petersburg, Russia) solution (polishing etchant) at room temperature. This etchant properly removes silicates [30], does not leave toxic reactants capable of interaction with BPE [31] and does not etch or oxidize silver [32]. The absence of a dielectric layer on the surface of SNPs is critical for SERS, for its presence diminishes or suppresses SERS and results in SNPs’ SPR spectral shift [33]. However it is worth mentioning that the latter phenomenon, in spite of decreasing SERS signal, is in use to tune the SPR wavelength [34], and covers allow protection of SNPs from contaminations [35].

## 3. Results and Discussion

**Figure 1:**
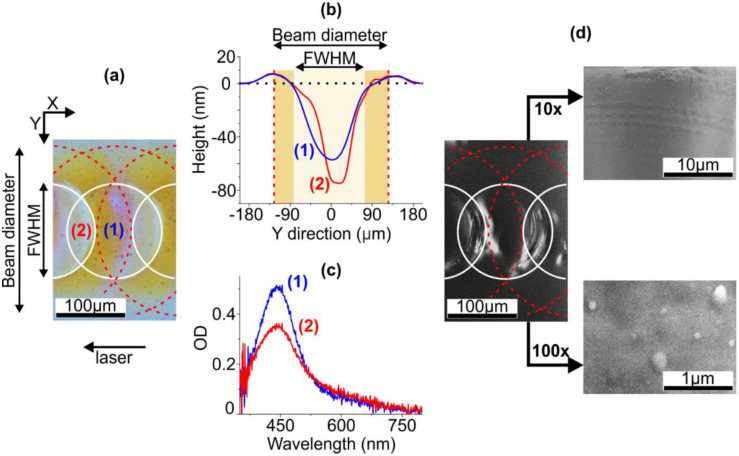
(**a**) Micrograph of the ion-exchanged glass irradiated with IR ns-laser, pulse energy 6.3 mJ, pulse frequency 2 Hz, the outlines corresponding to the laser beam diameter (dashed) and FWHM (solid) schematically show mutual position of the irradiation spots. The arrow shows the direction of the laser beam moving. (**b**) The profiles of the glass surface in regions (1) and (2) in the micrograph. (**c**) Local absorption spectra measured in the regions (1) and (2). (**d**) SEM images of the glass. Right panels: SEM images of higher resolution.

In the performed experiments, the IR ns-laser irradiation of the silver-ion-exchanged glass led to the formation of craters with elevated rims on the glass surface. The optical image of the sample surface with schematically outlined irradiation spots and the profile of the crater formed under the maximal pulse energy (6.3 mJ) are presented in Figure 1a,b, respectively. As seen, the depth of the crater is several tens of nanometers, and the diameter corresponds to the full width at half maximum (FWHM) of the irradiation beam, ~150 μm (outlined with solid lines in Figure 1a). The crater is surrounded by a broad, ~90 μm, elevated, <10 nm, rim placed along the perimeter of the irradiation spot. As one can see in Figure 1, in the regions where the FWHMs of the neighboring laser pulses overlap, a local increase in the crater depth is observed. The origin of the crater formation and deformation is in the pulse energy absorption by electrons, the energy being transferred to the glass network. This heating induces melting and partial sputtering (ablation) of the glass with the formation of a crater [36]. The thermocapillary and hydrodynamic forces act on the melt during its lifetime and induce the formation of an elevated rim and shock wave ripples around the crater [37].

The sample coloration indicates SNPs formation under the laser irradiation. As it was demonstrated [38], ns-laser interaction with soda-lime glass except heating results in the generation of non-bridging oxygen holes in the glass network, and in the case of silver-containing glasses this leads to reduction of silver ions and aggregation of silver atoms into NPs [3,14,17]. However, related studies provided no information neither about location of SNPs on or beneath the surface of the glasses nor SNPs distribution over the area affected by laser irradiation. To study the SNPs formation and distributions we characterized the laser-irradiated glass using absorption and Raman spectroscopy and scanning electron microscopy (SEM).

First, we studied the sample irradiated at 6.3 mJ and 2 Hz, the irradiation spots overlap being 50%, as-prepared. The local absorption spectra measured in colored (1) and transparent (2) regions of the sample are presented in Figure 1c. Note, that the colored region corresponds to the rim formed at the perimeter of the irradiation spot (see Figure 1a,b). The absorption peak appears at 450 nm, which corresponds to SPR wavelength in SNPs and indicates their formation, the maximal number of SNPs being at the perimeter of the irradiation spot. The drop of optical density and increase of the peak width in the transparent region (2) comparatively to the region (1) evidence not only a decrease in the number of SNPs, but also an increase in SNPs size dispersion in the region (2). One can assume uneven distribution of SNPs given by mutual position of the laser pulses. However, there are barely any SNPs in SEM images of the glass surface, see Figure 1d.

**Figure 2:**
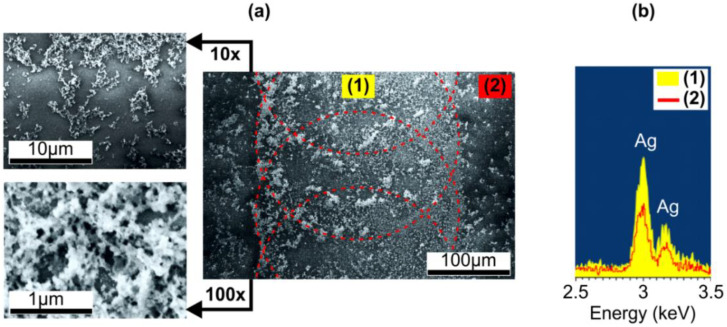
(**a**) SEM images of the ion-exchanged glass surface irradiated with IR ns-laser at 6.3 mJ and 2 Hz pulses after removal of ~25-nm glass layer via chemical etching. The outlines schematically show mutual position of the irradiation spots. Left panels: SEM images of higher resolution. (**b**) EDS spectra in (1) laser-irradiated and (2) non-irradiated regions on the glass surface after chemical etching.

An attempt to register Raman scattering enhancement (see Section 2) in the irradiated regions also gave no results. This allowed us to assumes that SNPs are buried under a dielectric (glass) layer. To remove this cover, the sample was etched in the polishing hydrofluoric etchant (see Section 2). The etching rate of the glass measured by the method of Ref. [39] was ~6 nm/min. The identical height difference between rims and craters before and after the etching allowed concluding that the etching rates of these regions coincided. The sample under study was etched for 4 min, i.e., ~25 nm of the glass was removed, and corresponding SEM image is presented in Figure 2a.

Comparing Figure 1d and Figure 2a, one can see that the contrast of the SEM images of the sample is improved after the etching. This improvement corresponds to less sample charging, i.e., higher surface conductivity, and, along with the surface topography modification, evidences the removal of SNPs’ cover by the etching. EDS measurements also demonstrated higher content of silver in laser-affected areas comparatively to non-irradiated regions on the sample after the etching (see Figure 2b). It should be noted, the coloring of the rim and the SPR absorption definitely indicate the input of metal nanoparticles in the EDS signal from the laser-irradiated area, while the EDS signal from the non-irradiated area, where no SPR was registered, should correspond to ionic silver content in the ion-exchanged glass. Observed SNPs are about 50 nm in size and aggregated into clusters. Notably, they are mainly at the periphery of the laser-affected area, where distinctly colored rim is (see dashed outline in Figure 2a). This confirms that SNPs participate in a mass flow induced by thermocapillary and hydrodynamic forces acting on a glass melt during its cooling after the irradiation as was reported for nanosecond pulses earlier [40]. In accordance with the Appendix A, the cooling time is significantly less than the pulse repetition interval, and the same way the growth of SNPs stops before the next pulse exposes the glass and it prolongs under the next pulse. Therefore, an increase in irradiation spots overlap should result in more uniform SNPs distribution in the laser-irradiated region.

**Figure 3:**
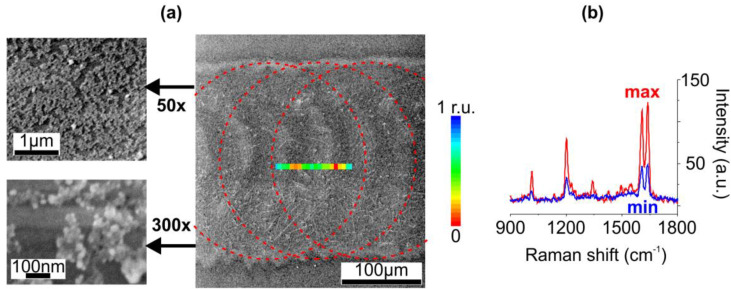
(**a**) SEM images of the ion-exchnaged glass surface irradiated with IR ns-laser at 5.6 mJ and 3.5 Hz pulses after removal of ~25-nm glass layer via chemical etching. The outlines schematically show mutual position of the irradiation spots. Inset: the map of the integral intensity of the 1200 cm^−1^ BPE Raman peak distribution. (**b**) Raman spectra in the areas of maximal and minimal enhancement in the map.

To obtain uniformly distributed SNPs we decreased energy of the pulses down to 5.6 mJ, decreasing materials’ sputtering, and increased their frequency up to 3.5 Hz, increasing the irradiation spots overlap up to 70%. The formed clusters, indeed, covered almost 90% of the laser-affected area, however, average size of SNPs in the clusters reduced down to 20 nm. Corresponding SEM images are presented in Figure 3a. We believe, this is because of secondary irradiation. The secondary irradiation of SNPs, as demonstrated in Ref. [41] for 532 nm ns-laser, leads to their fragmentation and partial removal from the surface. It is worth noting that the irradiation with both pulsed and continuous lasers can also result in the fragmentation and even dissolution of SNPs formed in ion-exchanged glasses [42,43].

Finally, after the etching, an intense SERS signal from deposited BPE was recorded. The Raman peaks in the measured spectra corresponded to vibrational modes of BPE [44]: 1010 cm^−1^ and 1600 cm^−1^ for pyridine rings breathing and stretching, 1200 cm^−1^ and 1640 cm^−1^ for C=C stretching. To compare sample topography with a SERS signal distribution we mapped the samples (see Section 2). As expected, the sample irradiated at the pulse frequency of 2 Hz provided Raman enhancement only at the periphery of the laser-affected area, where SNPs clusters were observed (the SERS map is not shown). The increase in the pulse frequency resulted in more uniform SERS signal distribution: the color map of the integral intensity of the 1200 cm^−1^ BPE peak distribution along the sample surface irradiated at 3.5 Hz imposed on the corresponding SEM image is in Figure 3a. The observed signal irregularities (see Figure 3b), however, may be attributed to SNPs aggregation.

The enhancement factor of the sample irradiated at the pulse frequency of 3.5 Hz was estimated using equation [45]:

(1) $$EF\;=\frac{I_{SERS}}{I_{Rs}}\cdot \frac{N_{RS}}{N_{SERS}},$$

where $I_{SERS}$ and $I_{RS}$ are intensities of a Raman peak measured from molecules absorbed on SERS-active and non-SERS-active substrates, respectively, and $N_{SERS}$ and $N_{RS}$ are numbers of molecules contributed to a corresponding signal. The 1200 cm^−1^ BPE Raman peak was used for quantification because of its insensitivity to BPE orientation on silver surface [46]. The ratio $\frac{I_{SERS}}{I_{Rs}}$ of the intensities integrated over FWHMs of the corresponding peak was ~1.5 for the spectra averaged over 20 measurements. The $N_{RS}$ was estimated by commonly used equation $N_{RS}=\rho^{BPE}h$ [47] with the known average thickness, h= 25 μm, and density, $\rho^{BPE}=$ 6.3 µmol/mm^3^, of BPE crystallites [48]. The accurate estimate of the $N_{SERS}$ in the center of the dried droplet, where SERS signal was collected, should account the mass flow of molecules toward the periphery of the droplet during its evaporation, so-called “coffee-ring” effect [49]. Therefore, we calculated the lower and upper estimates assuming the monolayer and the film of BPE molecules uniformly distributed over the area of the dried droplet, respectively. The surface concentration corresponding to the monolayer was established to be ~2 pmol/mm^2^, as the specific cell containing 2 BPE molecules in the case of the dense packing in the monolayer occupies ~1.6 nm^2^ [50]. The uniform BPE film was evaluated to be about 8 monolayers. Therefore, one can expect EF in the range ~10^4^–10^5^. We should note that the sample, irradiated at the pulse frequency of 2 Hz demonstrated an order of magnitude higher enhancement, the upper estimate to be ~10^6^. The obtained EF values are comparable with ones reported for SNPs prepared, for example, by laser ablation and thermal annealing of silver targets [51,52], fs-laser induced reduction from AgNO_3_ solution [53] and ns-laser modification of Ag@ZnO nanorods [54].

## 4. Conclusions

It is shown that IR nanosecond pulsed laser irradiation of silver-to-sodium ion-exchanged glasses results in the formation of glass-covered silver nanoparticles. ~25-nm deep etching removes the cover, and allows one to use uncovered nanoparticles to enhance Raman scattering from probed molecules. Surface distribution of nanoparticles and Raman enhancement depend on the overlapping percentage of the spots consecutively irradiated with laser pulses, e.g., 70% of the overlapping provides uniform distribution and up to ~10^5^ enhancement. The technique allows formation of given 2D SERS-active structures on the surface of the glass substrate and suitable for sensing applications.

## Appendix A

With the increase in the laser pulse energy at the fixed pulse frequency (3 Hz) from 5 to 6.3 mJ, we observed an increase in the crater depth, up to two times, as well as a slight (~1.4 times) increase in the crater diameter. In accordance with the obtained data, using the methodology [55], the sputtering (ablation) threshold was calculated, which amounted to ~3.2 mJ (7 J/cm^2^). Note that this threshold is several times less than the threshold for non-ion-exchanged glass [56]. We believe, it is because silver ions and clusters formed in the ion-exchanged glass behave as the source of seed electrons for avalanche ionization process and reduce the ablation threshold for nanosecond irradiation the same way as tin impurities in glasses do [56].

An upper estimate of the fraction of laser energy absorbed by soda-lime glass, the lifetime, and the thickness of glass layer melted because of the absorption of this energy without the account of lateral heat fluxes can be made using the following relations.

Equation (A1) for the ablation efficiency (fraction of absorbed energy) *θ* [57]:

(A1) $$\theta =\frac{\rho C_{\rho }\left(T_{v\;}-25℃\right)}{I/V}\approx 10^{-3},$$

where $\rho =2.5\;g/{\mathrm{cm}}^{3}$ density, $C_{\rho }=0.87\;\frac{J}{g℃}$ specific heat capacity, and *T_v_* = 2500–3500 °C evaporation temperature of the glass [58], *I* = 6.3 mJ the maximal laser pulse energy and *V* = 9 × 10^−10^ cm^3^ the volume of the crater formed as a result of the ablation at this energy.

Equation (A2) for the thickness of molten glass layer (*T_m_* = 720 °C the softening temperature of the glass [59]) at the average surface density of the maximal pulse energy $F=12\frac{J}{{\mathrm{cm}}^{2}}$ [60]:

(A2) $$h=\frac{\theta F}{T_{m\;}\rho C_{\rho }}\approx 80\;\mathrm{nm}.$$

Equation (A3) for the melt lifetime (before hardening):

(A3) t=h2D≈13 ns,

where *D* = 0.49 × 10^−6^ m^2^/s thermal conductivity of the glass [58].

The estimated lifetime of the melt is much shorter than the interval between the laser pulses, which allows us to state that the glass solidifies between consecutive pulses.
